# “Necessity is the mother of invention”: Experiences of accessing and delivering dementia-related support services by information communication technology during the pandemic in the UK

**DOI:** 10.1177/14713012241272906

**Published:** 2024-08-08

**Authors:** Thaïs Caprioli, Stephen Mason, Hilary Tetlow, Stan Limbert, Siobhan Reilly, Clarissa Giebel

**Affiliations:** NIHR ARC NWC, UK; Department of Primary Care and Mental Health, 4591 University of Liverpool, UK; Institute of Life Course and Medical Sciences, 4591 University of Liverpool, UK; NIHR ARC NWC, UK; Liverpool Service User Reference Forum (SURF), UK; NIHR ARC NWC, UK; 1905 University of Bradford, UK; NIHR ARC NWC, UK; Department of Primary Care and Mental Health, 4591 University of Liverpool, UK

**Keywords:** dementia, post-diagnostic support, support services, remote service delivery, pandemic, information communication technology

## Abstract

**Introduction:**

The remote delivery of dementia-related support services by information communication technology, defined as any hardware or software, including the telephone and videoconferencing software, increased during the coronavirus pandemic. To guide the future use of information communication technology, this study explored the experiences of delivering and accessing social care and support services during the pandemic in the UK.

**Method:**

Remote semi-structured interviews with social care and support providers, people with dementia and family carers were conducted between May-December 2022. Topic guides were co-developed with two public advisors (one former family carer, one person with dementia) and garnered information on delivering and accessing services during the pandemic. Audio recordings were transcribed verbatim. Employing a mixture of inductive and deductive analytic approaches, a thematic analysis was conducted.

**Results:**

Twenty-one interviews (*n* = 14 social care and support providers; *n* = 6 family carers; *n* = 2 people with dementia) were conducted. Three themes were generated: adapting to changing circumstances; responding to unmet needs by information communication technology and information communication technology should be a tool, not the default. Social care and support providers’ creativity and motivation facilitated the adoption of information communication technology, however, available resources and guidance varied. While some people with dementia and family carers benefitted from accessing services by information communication technology to address some needs, the format was not suitable for everyone.

**Conclusions:**

Beyond the coronavirus pandemic, the use of information communication technology within service delivery needs to be carefully considered, to avoid disenfranchising some people with dementia and family carers, while empowering people with the option of how to access services. Digital training and guidelines advising the use of information communication technology within service delivery may facilitate its improved use during the current landscape, and amidst future pandemics.

## Background

An estimated 920,000 people currently live with dementia in the UK, which is anticipated to rise to over a million by 2024 (Wittenberg et al., 2019). While substantial progress to facilitate an early diagnosis has been made, greater efforts to enable access to responsive post-diagnostic support are required (Arbalster & Brennan, 2022).

Post-diagnostic support includes services that respond to the care and support needs experienced by people with dementia and family carers, throughout the disease trajectory (Prince et al., 2016). Within post-diagnostic support, social care and support services can be defined as non-clinical services that support community-dwelling people with dementia, and family carers, to live well. In the UK, social care and support services are provided by multiple sectors, providers and settings. Services are largely provided by local authorities and Third Sector organisations, and services may include paid home care, befriending services, daycare centres and respite care. Social care and support services are important to living well with dementia, and may promote independence, facilitate meaningful engagement and alleviate the family carer burden (Camic et al., 2013; Kelly & Innes, 2016; Willis et al., 2018). Nevertheless, access to social care and support services can be problematic, and persisting unmet needs are unequally distributed (Bieber et al., 2019; Ketchum et al., 2023).

The coronavirus pandemic and associated public health measures engendered significant changes to service delivery, notably during the height of the pandemic when widespread physical distancing was enforced across the UK (Institute for Government, 2022). Owing to the closure of in-person services, social care and support services shifted to deliver remote or hybrid services (Caprioli, Giebel, et al., 2023), often by using information communication technology. Here, we conceptualised information communication technology as any hardware or software, such as telephone calls, emails and videoconferencing software, to remotely access social care and support services. The coronavirus pandemic increased, at least temporarily, the reliance on information communication technology, and presents a unique opportunity to explore the barriers and facilitators to delivering and accessing social care and support services by information communication technology.

Spanning from managerial to frontline positions, social care and support providers experienced significant changes in working practices during the coronavirus pandemic. Several social care and support organisations established welfare calls (O'Rourke et al., 2021), and employed videoconferencing software to deliver live or pre-recorded support (Cousins et al., 2022; Lee et al., 2021; Masoud et al., 2021; Wheatley et al., 2022). Reactively turning towards information communication technology enabled solutions is likely to have involved numerous challenges, including tailoring the content of services to suit a remote format and identifying suitable approaches to assist people with dementia access services, often by working with family carers (Cowley et a., 2023; Di Lorito et al., 2021). The pandemic engendered substantial hardship among people with dementia and family carers (Giebel et al., 2023b, 2023a), and some post-diagnostic providers adjusted by delivering greater emotional support (Wheatley et al., 2022). Documenting how social care and support providers employed information communication technology to respond to people with dementia and family carers’ needs may help the preparedness of service delivery amidst future public health crises.

Concerningly, fewer people with dementia and family carers accessed social care and support services during the pandemic (Giebel et al., 2021). While accessing post-diagnostic support remotely appears feasible for some people with dementia and family carers (Caprioli, Mason, et al., 2023), access often hinges on the availability of devices, reliable connectivity, adequate digital literacy and/or the opportunity to receive support to navigate the technology (Arighi et al., 2021; Robinson et al., 2015, 2020; Yi et al., 2021). The shift to remote social care and support services delivered by information communication technology during the coronavirus pandemic was welcomed by some people with dementia and family carers, and remote services may provide several benefits, including convenience (McLoughlin et al., 2023) and bypasses barriers associated with rural residency (Bayly et al., 2020). Nevertheless, some people with dementia and family carers experienced difficulties while navigating technology due to limited digital literacy and/or while engaging with services remotely (Caprioli, Mason, et al., 2023; Gerritzen et al., 2023), with some preferring to access post-diagnostic support in-person (Caprioli, Mason, et al., 2023). Exploring people with dementia and family carers’ experiences of using information communication technology to access an array of social care and support services may help guide its future use within service delivery.

Thus, this study sought to (1) understand how information communication technology has been employed to deliver remote social care and support services during the coronavirus pandemic and (2) explore the experiences of delivering and accessing social care and support services by information communication technology during the coronavirus pandemic in the UK.

## Methods

This study builds on our previous survey, addressed to social care and support providers, that compared the types of services delivered and delivery methods employed prior to and during the coronavirus pandemic in the UK (Caprioli, Giebel, et al., 2023).

Here, grounded by a critical realist epistemological stance, a qualitative study design was applied to explore the experiences of people with dementia, family carers, and social care and support providers (Gill et al., 2008). Critical realism adopts a realist ontology and a constructivist epistemology (O’Mahoney & Vincent, 2014), and enabled the contextualised exploration of the experiences of delivering and accessing social care and support services.

### Participants and recruitment

Social care and support providers were defined as those who deliver non-clinical community-based social care and support services for people with dementia and/or family carers, either in a paid or volunteering (unpaid) capacity, in any sector (public, private or Third sector). Social care and support services includes an array of services, including peer support groups, information provision, and social activities. Participants were eligible if they delivered social care and support service prior to and during the pandemic in the UK and were above the age of 18 (Table 1). Social care and support providers were recruited from participants from our previous survey (Caprioli, Giebel, et al., 2023) who expressed interest in future research. In our survey, social care and support providers were recruited by convenience sampling (no gatekeepers involved), across the UK, through several avenues, including contacting publicly available email addresses, engaging with social prescribing routes, and sharing the research opportunity on social media.

**Table 1.** Eligibility criteria for each study population.

Social care and support providers	People with dementia	Family carers
(1) Currently working (paid capacity) or helping/volunteering (unpaid capacity) to deliver social care and support services within the public, private or third sector in the UK; (2) delivering social care and support services to people with dementia and/or to family carers living in the community; (3) either directly involved in delivering social care and support services or involved in the management/organisation of the delivery of services and (4) at least 18 years of age	(1) Lives in the community; (2) accessed social care and support services in the UK prior to and during the COVID-19 pandemic; (3) has mental capacity; and (4) at least 18 years of age	(1) Accessed social care and support services prior to and during the COVID-19 pandemic in the UK that either addresses their needs, the needs of the person with dementia or the dyad’s needs; and (2) at least 18 years of age

Here, we endeavoured to engage a maximum variation sampling approach (Green & Thorogood, 2018) to the recruitment of social care and support providers. The following previously collected elements guided the invitation of social care and support providers: socio-demographic factors; Index of Multiple Deprivation quintile of service provision area (Department for Communities and Local Government, 2015); geographical location; sector and types of services delivered; and digital and non-digital information communication technology formats employed. However, owing to challenges to recruit, the research opportunity was shared with all eligible social care and support providers (*n* = 43).

People with dementia who were deemed to have the mental capacity to participate, and current and/or former family carers were eligible if they resided in the community and had accessed social care and support services in the UK prior to and during the pandemic (Table 1). People with dementia and family carers were recruited by convenience sampling. The research opportunity was shared on X (formerly Twitter), at in-person dementia events and amongst known social care and support organisations.

### Data collection

Interviews were conducted remotely by Zoom or telephone between May and December 2022. Two semi-structured topic guides were co-developed with public advisors and the wider research team. Topic guides garnered information on delivering and accessing social care and support services prior to and during the pandemic. A public advisor (HT) with experience in delivering and accessing social care and support services prior to and/or during the pandemic was involved in the piloting of the topic guides.

TC conducted all interviews. Interviews with social care and support providers were conducted on a one-to-one basis and depending on people with dementia and family carers’ preferences, interviews were offered on a one-to-one basis or as a dyad. Verbal informed consent was obtained and recorded prior to interview. To assess the ability of potential participants with dementia to make an informed decision regarding their participation, individuals were asked to summarise their understanding of the study and reasons for participating in accordance with the Mental Capacity Act (2005). TC conducted all mental capacity assessments, and CG, an experienced dementia researcher, was present during all assessments. Field notes were recorded following each interview to assist ongoing reflexivity. TC transcribed a third of the interview audio-recordings verbatim, and the remainder were transcribed by a professional agency from the University of Liverpool.

To characterise our sample of people with dementia and family carers, age, gender, ethnic background, and if applicable, the dementia diagnosis was collected.

### Data analysis

We undertook a deductive and inductive thematic analysis to yield descriptive and interpretative accounts of using information communication technology to deliver and access social care and support services. Following the six phases of thematic analysis (Braun & Clarke, 2006), transcripts were read multiple times. Subsequently, using a mixture of inductive and deductive analytic approaches, TC and manually coded all transcripts. One coded transcript was discussed with CG, and two coded transcripts with HT. To assess the referential competency of the analysis, the pilot themes and sub-themes were compared to the raw data (Nowell et al., 2017). A pragmatic 10% of transcripts from each study population were compared to the pilot analysis. Final themes and sub-themes were collectively agreed upon.

### Public involvement

Two public advisors, a former family carer and a person living with dementia, were involved throughout the study, and have experience in delivering and accessing social support services prior to and/or during the pandemic. HT undertook training on coding and coded two transcripts. HT and SL attended meetings to design the study, interpret the findings and were involved in writing a lay summary of the findings.

### Ethical considerations

Ethical approval was granted by the University of Liverpool (Reference: 10786) prior to the commencement of the study.

Several approaches were undertaken to support people with dementia to participate in our study. Guidance from The Dementia Engagement and Empowering Project (DEEP, 2013) was followed while writing the study information sheet, and the document was reviewed by the public advisors. The mental capacity of potential participants with dementia to participate in our study was assessed prior to interview, and people with dementia had the opportunity to invite a family carer to participate in the study with them. Interviews were scheduled at a time that was convenient to participants, and during the interviews, TC paid attention to signs of fatigue or upset. If any were identified, the interview paused and only continued if participants were happy to do so. Two debriefing sheets were devised, one for social care and support providers, and one for people with dementia and family carers, and shared if participants were distressed.

## Results

Twenty-one interviews were conducted (*n* = 14 social care and support providers; *n* = 2 people with dementia and *n* = 6 family carers^1^) and lasted a mean of 32 minutes (range:11–60). All participants were interviewed on a one-to-one basis by videoconferencing software, except for one social care and support provider and one family carer who participated on a one-to-one basis by telephone.

Most social care and support providers identified as female and worked in a paid capacity within the Third Sector (Table 2). All family carers identified as White and female. Most were aged between 55–64 years (*n* = 5 (83.3%)), with one family carer aged above 65. All people with dementia identified as White and male, with one participant aged between 55–64 years, and one above 65. One participant was diagnosed with mixed dementia and dementia with Lewy bodies and one participant was diagnosed with Lewy body and Parkinson’s Disease dementia.

**Table 2.** Socio-demographic characteristics of the social care and support providers (*n* = 14).

Social care and support providers *n* (%) *n* = 14	
Gender	
Female	10 (71.4)
Male	4 (28.6)
Age group	
35–44	3 (21.4)
45–54	3 (21.4)
55–64	4 (28.6)
65 and over	4 (28.6)
Ethnicity	
White	13 (92.9)
Mixed/Multiple	1 (7.1)
Sector	
Public	1 (7.1)
Private	2 (14.3)
Third sector	11 (78.6)
Contractual arrangement	
Paid	9 (64.3)
Volunteer	5 (35.7)
Region	
East of england	3 (21.4)
Greater London	1 (7.1)
North West	5 (35.7)
South east	2 (14.3)
West Midlands	1 (7.1)
Scotland	2 (14.3)
IMD quintile of service provision area^a^	
1	2 (14.3)
3	4 (28.6)
4	3 (21.4)
5	3 (21.4)

^a^These represent the IMD quintiles of areas in England. Two participants provided services in Scotland, in areas classified in the third quintile (Scottish Index of Multiple Deprivation, 2020).

We generated three overarching themes: (1) adapting to changing circumstances; (2) Responding to unmet needs by information communication technology; and (3) information communication technology should be a tool, not the default. Table 3 identifies which study population group relates to each sub-theme.

**Table 3.** Table depicting the themes and sub-themes and which population group they relate to.

Theme and sub-themes	Study population
1. Adapting to changing circumstances	
1.1 going the extra mile	Social care and support providers
1.2 characterising guidance and resources	Social care and support providers
1.3 supporting staff and volunteers by information communication technology	Social care and support providers
2. Responding to unmet needs by information communication technology	
2.1 basic needs	Social care and support providers. The needs experienced are reported by people with dementia and family carers interviewed and/or observed by social care and support providers
2.2 alleviating stress and loneliness	Social care and support providers. The needs experienced are reported by people with dementia and family carers interviewed and/or observed by social care and support providers
2.3 implications to practice	Social care and support providers
3. Information communication technology should be a tool, not the default	
3.1 inequalities in access to information communication technology devices and digital literacy	Experiences of people with dementia and family carers interviewed and/or those observed by social care and support providers
3.2 (Un) suitability of accessing social care and support services by information communication technology	Experiences of people with dementia and family carers interviewed and/or those observed by social care and support providers
3.3 views on the future use of information communication technology in service delivery	Social care and support providers

## Theme 1: Adapting to changing circumstances

### Going the extra mile

Social care and support providers’ motivation and creativity facilitated the delivery of timely, personalised and flexible support by information communication technology. Through an ongoing process of trial and error and seeking input from people with dementia and family carers, social care and support providers found the confidence to implement new ideas to adapt service delivery to reflect people with dementia and family carers’ needs, preferences and digital abilities. Examples included the delivery of support by digital and non-digital media, convincing senior management to establish a weekend helpline and checking in on family carers, by telephone calls, if they did not attend groups delivered by videoconferencing software.

‘I mean we were doing it, but it [singing sessions by videoconferencing software] got better all the time. So, we had to learn how to do it effectively…’ Social care and support provider, Third Sector (P3)

‘… not all of our members initially wanted to engage [in activities delivered by videoconferencing software (Zoom)] … so that was when we implemented the telephone calls to make sure that people weren’t left in isolation and forgotten about’ Social care and support provider, Third Sector, (P13)

### Characterising guidance and resources

Guidance received varied among social care and support providers, with some receiving little guidance to deliver services by information communication technology. However, many social care and support providers enjoyed the creativity required to adapt services, and in the absence of guidance, some social care and support providers and/or their organisation created internal procedures and/or sought advice from universities or other social care and support organisations.

‘…[the] organisation’s approach was they set up very quickly a policy for conducting welfare calls…’ Social care and support provider, Third Sector (P4)

‘… we didn't really have any support as organisers we just got on with it…’ Social care and support provider, Private Sector (P6)

The types of resources varied across social care and support providers and organisations. The availability of funds differed, which seemed, to some extent, impact the ability to equip social care and support providers with information communication technology devices and/or lend digital devices to people with dementia and family carers. Some social care and support providers received grants to cover the cost of lending tablets (including dementia-specific tablets) to people with dementia and family carers.

‘…we, um very quickly, started to get some support grants coming in …we bought some tablets, we got some tablets with um SIM [subscriber identity module] cards built in and just brought a data plan for a year for people…’ Social care and support provider, Third Sector (P5)

‘That made life much easier because all of the staff of the day service had laptops…’ Social care and support provider, Public Sector (P14)

Moreover, the regularity of access to information communication technology devices and level of digital literacy varied across social care and support providers. Some social care and support providers independently delivered services by videoconferencing software while home working and/or relied on the assistance of colleagues; however, others relied on external stakeholders or delivered services by telephone. For some social care and support providers, newly or previously forged partnerships with external stakeholders were important to access information communication technology devices and/or receive digital support. External stakeholders included a library and other social care and support organisations.

‘…we would rely on the library to [do] the calls for the telephone singing the group telephone singing because we don't have… I don't know how you get [Microsoft] Teams [videoconferencing software] … but I imagine it's megabucks and we don't have an income' Social care and support provider, Third Sector (P11)

Furthermore, many social care and support providers re-allocated their volunteers to help deliver services by information communication technology during the pandemic, which included conducting befriending calls by telephone.

‘We had volunteers work in teams and provide a kind of telephone befriending service, checking whether they needed any shopping or anything’ Social care and support provider, Third Sector (P9)

### Supporting staff and volunteers by information communication technology

Reactively shifting to deliver services by information communication technology instilled stress amongst social care and support providers, with one provider reporting to have experienced burnout. Social care and support providers were simultaneously bearing the impact of the pandemic professionally and within their personal lives, and access to wellbeing support within the workplace seemed limited.

‘…bang straight working from home and the next days and you know and then I'm thinking right I need to ensure I can provide for my children and make sure they're safe for lockdown' Social care and support provider, Third Sector (P13)

Nevertheless, some social care and support providers established wellbeing initiatives for their staff and volunteers. These took form as regular telephone calls and/or social activities by videoconferencing software. Upon reflection, many social care and support providers would place greater efforts in promoting staff and volunteer well-being.

## Theme 2: Responding to unmet needs by information communication technology

### Basic needs

The term ‘basic needs’ was conceptualised to refer to food, shelter and safety. When public health measures were most restrictive, society shifted towards digital spaces, and access to information communication technology devices and digital literacy often formed prerequisites to fulfilling one’s basic needs. While some people with dementia and family carers required little support to address their basic needs, some faced significant challenges.

‘We were you know, struggling for food, struggling for everything really’ Family carer to her husband (C5)

‘…we were pretty self-sufficient, you know we got into a routine…’ Former family carer to her mother (C4)

To this end, employing a mixture of proactive (e.g., welfare telephone calls) and reactive approaches (e.g., telephone helplines), social care and support providers were informed of, and able to respond to people with dementia and family carers’ needs. Responding to needs included remotely liaising with relevant stakeholders, including local councils and supermarkets, to organise food deliveries.

‘… I rang them [people with dementia and/or family carers] up to do a welfare call to see how they were doing I had to then go off and with the local authority and organise food' Social care and support provider, Third Sector (P4)

Moreover, some social care and support providers responded to safety concerns, which included disseminating online safety advice regarding the fluctuating coronavirus public health measures and supporting suicidal family carers by telephone. The latter was challenging, and on critical occasions, one social care and support provider reported that in-person support was deemed necessary.

‘When you've got a [family] carer phoning up saying I'm going to kill myself then we've kind of got to say well actually we're got to go we've got to go and do something face to face with them' Social care and support provider, Third Sector (P8)

### Alleviating stress and loneliness

From the sudden withdrawal of social care and support services during the initial phase of the coronavirus pandemic, to living under lockdown, feelings of isolation and loneliness were abundant amongst people with dementia and family carers.

‘Everything just folded around us. It was frightening really, because… apart from phone concept, contact, that was it [interviewer's name]. Just gone. In a flash because of the legal constraints’ Person with dementia (PD2)

‘I was living on my own and couldn’t go anywhere you know. It was just horrendous, it really was.’ Family carer to her husband (C1)

Family carers felt particularly isolated, notably if the person they were caring for became non-verbal or had died. Many family carers undertook additional caring hours with limited or no respite which instilled stress, and some navigated challenging decisions regarding the admission of the person they cared for to a care home.

‘It’s not the time I would want to live through again, especially having to care 24/7, as well as keep him safe from COVID [coronavirus infection] and keep myself safe from COVID [coronavirus infection], and [takes a deep breath] it was just the worst time I think ever.’ Family carer to her husband (C5)

To increase opportunities to socialise, some social care and support providers established WhatsApp (an electronic messaging service) groups, sent newsletters by email, and organised peer support groups and social activities by videoconferencing software (Zoom and Microsoft Teams) and Facebook Live. One social care and support provider loaned tablets and remotely assisted two people with dementia to navigate the technology, enabling them to independently remain in contact.

‘… and they'd all moan or chat to each other or tell each other what they were doing and then they got, gradually got the hang of it [WhatsApp] and they put pictures of what their garden was like and all the things that they were doing that was really nice' Social care and support provider, Private Sector, (P6)

In addition to synchronous support, some social care and support providers delivered support asynchronously. This included pre-recorded singing sessions, videos to help de-stress and to promote exercise health. Some asynchronous videos were shared by email, and/or available on YouTube or on a library’s website.

‘We also recorded a few of our own in house erm so like singing or different activities that people could then, we put onto YouTube so people could go to them at any time' Social care and support provider, Third Sector (P8)

### Implications to practice

Social care and support providers undertook several measures to increase people with dementia and family carers’ access and engagement with remote services. To bridge limited digital device ownership, some social care and support providers loaned tablets to people with dementia and family carers’ (see subtheme characterising guidance and resources). Prior to lending tablets, the devices were rendered more user-friendly, notably to facilitate the use of videoconferencing software (Table 4). However, assisting people with dementia and family carers to navigate videoconferencing software by telephone was often challenging. Owing to the limited capacity to deliver training remotely, one social care and support provider avoided applying for funds to lend tablets, and another delivered socially distanced training at people with dementia and family carers’ doorstep.

‘…but literally took me two hours, just to get her to turn the device on, because, she was turning it on, and then instantly turning it back off again. She just got into this loop’ Social care and support provider, Third Sector (P5)

‘…because that was difficult over the phone trying to explain to people oh if you press this button, well I can't see that button’ Social care and support provider, Third Sector (P8)

**Table 4.** A summary of practical suggestions to facilitate engagement and access to services delivered by videoconferencing software.

Preparing digital devices
- Downloading and installing videoconferencing software beforehand
- Increasing font size
- Devising device specific manuals
- Locking the emplacement of apps on home screen
- Extending screen automatic lock-out times
- Removing passwords
Facilitating engagement
- Sending multiple reminders
- Providing a schedule of online activities and/or sharing the schedule of online activities provided by other organisations
- Communicating themes/topics to be discussed and/or sending song lyrics (by post or email) beforehand
- Maintaining a regular schedule
- Mirroring the schedule employed while delivering in-person services
- Pre-recorded videos (shared by email and web pages)

Moreover, to remove difficulties with navigating new information communication technology, one social care and support provider delivered singing sessions by Microsoft Teams (videoconferencing software), which enabled people with dementia and family carers to access sessions from their telephones. To facilitate engagement with services delivered by videoconferencing software, some social care and support providers sent lyrics, by email and/or post, ahead of sessions (Table 4).

## Theme 3: Information communication technology should be a tool, not the default setting

### Inequalities in access to information communication technology devices and digital literacy

People with dementia and family carers’ access to information communication technology devices varied, with some social care and support providers reporting financial barriers.

‘…people, people who couldn’t afford the technology were really really adversely impacted.’ Social care and support provider, Third Sector (P2)

Social care and support providers observed that people with dementia and family carers were most familiar with landline telephones, and identified that, while many had email addresses, internet connectivity and access to digital devices, ownership did not always translate to the ability to access services remotely. Barriers observed by social care and support providers and/or reported by people with dementia and family carers included a lack of confidence, fear of breaking devices and ownership of outdated devices that were incompatible with recent videoconferencing software. Nevertheless, either independently and/or with support from family carers, some people with dementia were able to navigate information communication technology, including videoconferencing software, to access support remotely.

‘…at first, I-I was a bit frightened about it [videoconferencing software] … but when I-I got on to it, I-I found it really interesting because b-being able to contact people and stuff like that, and…’Person with dementia (PD1)

‘So I sort of had to have all the buttons on the computer [chuckles] if not he’d [husband with dementia] press it, and we’d lose something!’ Family carer to her husband (C7)

Some social care and support providers observed the heterogeneity in family carers’ digital literacy, and most family carers interviewed were cognisant of their fortunate position, attributing their skills to previous employment.

‘I think it must be very difficult if you haven’t got any computer skills or if you are isolated that must be a nightmare because you would really feel so alone you know’ Family carer to her mother (C3)

### (Un) suitability of accessing social care and support services by information communication technology

The visual cues offered by videoconferencing software was valued by people with dementia interviewed, however, some social care and support providers and family carers observed that videoconferencing software proved to be a sensory overload, confusing and/or minimally engaging for some people with dementia. Moreover, some social care and support providers observed that some people with dementia found it easier to engage with one-to-one services delivered by videoconferencing software, whereas others preferred group-based formats.

‘… [videoconferencing software] better than phone call… Cos you can actually see a person then’ Person with dementia (PD1)

‘Um… wasn't as good for [husband with dementia] because it’s very flat, isn't' it? You know, um he still will look at a Zoom call, um… he does go on Zoom with me, but he doesn't like it as much as seeing somebody in the room.’ Social care and support provider, Third Sector and family carer to her husband (P1)

Social care and support services delivered by information communication technology were unable to replicate the companionship experienced and valued when accessing services in-person but produced many benefits in the absence of this option. These included helping to combat feelings of loneliness, facilitating access to people who were housebound and connecting people with dementia and family carers with others across the UK. Furthermore, the use of videoconferencing software proved to be a valued medium to continue to access social care and support services when the coronavirus public health measures were removed, and anxiety regarding re-engaging in in-person services was high.

‘…somebody said it [singing sessions by videoconferencing software] made her [person with dementia] feel, more lonely that ever. Because in normal singing [in-person format] she'd be able to turn around and smile at someone or put her around them, whereas you couldn't do that…’ Social care and support provider, Third Sector, (P11)

‘I am still a bit wary about meeting up in person with people mainly because I want to keep safe so that I can still see my husband [who lives in a care home]’ Family carer to her husband (C1)

Social care and support providers observed that, overall, family carers seemed to garner more benefits from accessing social care and support services by information communication technology than people with dementia. However, people with dementia interviewed, reported to have greatly benefitted from accessing peer support groups by videoconferencing software.

‘…if I had a choice, I would go back to one-to-one [in-person], but without technology [videoconferencing software] I would have been in a very different place emotionally and physically…’ Former family carer to her mother, current family carer to her mother-in-law (C2)

‘When we started to use electronic methods like this [videoconferencing software], visually and audially, and face-to-face, it brought me help. It brought me out of the depression. This-this is fantastic.’ Person with dementia (PD2)

Some people with dementia and family carers reported negative experiences of accessing social care and support services by information communication technology. These seemed to relate to wider barriers in access, including the appropriateness, times and/or frequency of services delivered during the coronavirus pandemic, rather than due to accessing services by information communication technology.

‘I think just the occasional phone call when they call you, I mean it did happen but maybe more structure around that and more regular’ Former family carer to her mother (C4)

‘I mean there are groups available but again it’s the timing of them, they all seem to be in the mornings or there was one I think 12 – 1 again I can’t make it because the carers are back then so yes’ Family carer to her mother (C3)

### Views on the future use of information communication technology in service delivery

Social care and support providers’ views towards using information communication technology within future service delivery were mixed. While some recognised the value information communication technology may offer when complementing in-person services, some social care and support providers opposed its use. One social care and support provider considered the possibility of delivering services exclusively by information communication technology on a short-term basis, during adverse weather or illness.

‘…it’s possible that-that the people receive lots of different ideas and toolkits and resources [by videoconferencing software] to help them. But, but honestly, it needs scaffolding by real people in the room’ Social care and support provider, Third Sector (P2)

‘To me it's not the way forward but that might be my grey hairs.' Social care and support provider, Third Sector (P3)

Furthermore, the use of information communication technology may provide non-service recipient facing benefits. The pandemic digitalised some elements of the internal working of two organisations, with one social care and support provider reporting that the introduction of a WhatsApp group among staff and volunteers facilitated prompt decision-making.

‘It [WhatsApp group] was instant decisions which was to be honest with you when we when we have our meetings it very very slow and very very considered and very very cautious... now it was bang bang bang lets do this' Social care and support provider, Third Sector (P13)

## Discussion

This study adds to the evidence-base by exploring how information communication technology has been employed to deliver and access social care and support services during the coronavirus pandemic and provides insights to guide its future use within service delivery. Social care and support providers demonstrated resilience while adapting to delivering services by information communication technology, however, many lacked guidance and resources. While the reliance on information communication technology benefitted some people with dementia and family carers, it excluded others. Moving beyond the pandemic, using information communication technology to complement in-person services may help to avoid the widening of inequalities in access, while empowering people with dementia and family carers with the choice of how support is accessed.

We found that the use of information communication technology within service delivery was guided by an ongoing process of trial and error, sustained by social care and support providers’ creativity and motivation. Akin to therapists delivering an exercise intervention remotely during the pandemic (Cowley et al., 2023; DiLorito et al., 2021), social care and support providers interviewed appreciated the creativity required to reactively adapt services towards information communication technology enabled solutions. Social care and support providers’ creativity may benefit service innovation beyond the pandemic (DiLorito et al., 2021), which is important to the sustainability of services, and forms a key pillar in the governments’ social care reform White paper (Department of Health and Social Care, 2021).

However, fostering social care and support providers’ creativity will only go so far, and organisations’ information communication technology capacity and infrastructure are instrumental. Our analysis identified considerable heterogeneity in terms of digital literacy amongst social care and support providers. Indeed, opportunities to access digital training (Chapman, 2022), equipment/devices and the availability of guidelines may be influenced by the size of the social care and support organisation, and the sector it operates in. Equipping social care and support providers, including volunteering providers, with digital skills may benefit their career progression (Blake et al., 2021), facilitate the improved use of information communication technology to deliver services, and strengthen their ability to assist people with dementia and family carers to access services remotely. This is important as the latter has been identified as a facilitator to increase people with dementia and family carers’ access to remote services (Yi et al., 2021). Furthermore, the availability of guidelines to help deliver services by information communication technology is likely to promote standardisation and benefit social care and support providers, people with dementia and family carers. For example, when comparing the implementation of safe long-term care visitation guidance in the Netherlands during the height of the coronavirus pandemic to the lack of guidance in the UK, Giebel and colleagues (Giebel et al., 2023c) found that standardised guidance benefited providers, residents and their families.

Aligning with previous studies (Gerritzen et al., 2023; Masoud et al., 2021; McLoughlin et al., 2023), our findings suggest that people with dementia and family carers’ ability to remotely access and engage with social care and support services delivered by information communication technology varied. Limited access to information communication technology devices, internet connectivity and/or poor digital literacy, which are often influenced by socio-economic factors (Robinson et al., 2015, 2020), are likely to have contributed to the heterogeneity observed in our study. Moreover, technical glitches (Lee et al., 2021; Vellani et al., 2022) and dementia-related factors, including difficulties in following conversations remotely (Gerritzen et al., 2023, 2024), may result in additional challenges to engaging with services delivered by information communication technology. The use of videoconferencing software, compared to telephone calls, was percieved as the closest substitute to accessing an in-person advanced care planning intervention by some people with dementia and family carers (Vellani et al., 2022). Moreover, accessing peer support by videoconferencing software enabled people with dementia to feel socially connected, however, many missed the physical gestures of support, including hugs and pats on the back, made possible when attending peer support groups in person (Talbot & Briggs, 2022). Nevertheless, services delivered by information communication technology can offer numerous benefits, including convenience (Masoud et al., 2021; McLoughlin et al., 2023), rendering services more accessible (Talbot & Briggs, 2022) and enabling people with dementia to access support in a format that aligns with their preferred communication method and/or needs (by text or verbally) (Gerritzen et al., 2023). Furthermore, akin to Gerritzen and colleagues (2024), who found that some people with young-onset dementia found it challenging to identify remotely delivered peer support groups that reflected their needs and/or interests, our analysis suggests that the consideration of wider barriers in access, including the appropriateness and service delivery times, are important to facilitate access to remote social care and support services.

## Strengths and limitations

This study adds to the evidence-base by exploring how information communication technology has been employed to deliver social care and support services remotely during the coronavirus pandemic in the UK. Two public advisors were involved throughout, which strengthened the development of the topic guides and the interpretation of the findings. However, several limitations are acknowledged. Most social care and support providers delivered services within the Third Sector and served relatively affluent areas (based on Index of Multiple Deprivation quintile of service provision areas). Thus, our findings may not be representative of how information communication technology was employed within service delivery across sectors and/or organisations serving more deprived areas. Furthermore, it is possible that our sample consists of social care and support providers whose services optimally transitioned to information communication technology enabled service delivery, and we did not collect information on whether social care and support providers undertook frontline and or managerial duties, which is likely to have influenced their experiences. Lastly, while several recruitment avenues were undertaken and the data collection timeframe was extended, the small size of people with dementia and family carers is a limitation. The data collection timeframe was not further extended due to resource constraints. Nevertheless, the small sample size of people with dementia and family carers is complemented by the proxy accounts provided by social care and support providers.

## Conclusions

Accessing services remotely by information communication technology may benefit some people with dementia and family carers but may disenfranchise others. Thus, to empower people with dementia and family carers with the choice of how services are accessed, the use of information communication technology within service delivery requires careful consideration. Social care and support providers’ motivation and creativity facilitated the use of information communication technology within service delivery during the coronavirus pandemic, however, access to resources and guidance varied. Digitally training social care and support providers and devising guidance on delivering services by information communication technology may facilitate its improved use within the current hybrid landscape, and amidst future public health crises.

## Note

1. One participant was a social care and support provider and a family carer.
